# The 21st Century Cures Act: A Competitive Apps Market and the Risk of Innovation Blocking

**DOI:** 10.2196/24824

**Published:** 2020-12-11

**Authors:** William J Gordon, Kenneth D Mandl

**Affiliations:** 1 Department of Medicine Brigham and Women's Hospital Boston, MA United States; 2 Mass General Brigham Boston, MA United States; 3 Harvard Medical School Boston, MA United States; 4 Computational Health Informatics Program Boston Children's Hospital Boston, MA United States

**Keywords:** interoperability, application programming interfaces, SMART on FHIR, health information, patient records, digital infrastructure, digital, app ecosystem

## Abstract

The 21st Century Cures Act and the recently published “final rule” define standardized methods for obtaining electronic copies of electronic health record (EHR) data through application programming interfaces. The rule is meant to create an ecosystem of reusable, substitutable apps that can be built once but run at any hospital system “without special effort.” Yet, despite numerous provisions around information blocking in the final rule, there is concern that the business practices that govern EHR vendors and health care organizations in the United States could still stifle innovation. We describe potential app ecosystems that may form. We caution that misaligned incentives may result in anticompetitive behavior and purposefully limited functionality. Closed proprietary ecosystems may result, limiting the value derived from interoperability. The 21st Century Cures Act and final rule are an exciting step in the direction of improved interoperability. However, realizing the vision of a truly interoperable app ecosystem is not predetermined.

## Introduction

In May 2020, the Department of Health and Human Services and the Office of the National Coordinator for Health Information Technology (ONC) published a final rule implementing health information technology provisions of the 21st Century Cures Act [1]. The rule defines standardized methods for obtaining computable, electronic copies of electronic health record (EHR) data through an application programming interface (API). APIs enable granular, computable, immediate access to data and allow patients or provider organizations to connect an app directly to the EHR that exchanges data without an intermediary. The rule standardizes the Fast Healthcare Interoperability Resources (FHIR) data model and the Substitutable Medical Applications, Reusable Technologies (SMART) on FHIR standard [2,3], which specifies how users are authorized and how apps launch. The rule restricts providers and EHR vendors from “information blocking” — preventing the exchange of electronic health information.

Intense lobbying against the rule, preceding its release, by EHR vendors and hospital systems signals that control over data and revenue are both at stake. The rule not only enables patient and provider access to medical record data in computable formats but also shifts how they interact with the health care system, which entities control those interactions, and the underlying business models that could either threaten or accelerate interoperability’s potential to improve care delivery.

## Supporting an Interoperable App Economy

The rule is designed to foster a competitive ecosystem of substitutable (often third-party) apps that can be written once and connect to standardized health system data anywhere. The Cures Act requires an API that makes “all data elements” of a patient’s record available “without special effort” [4]. Examples of apps that leverage such an API include Apple’s Health Records on iPhone product, which enables patients to download computable copies of their medical records from hundreds of health systems [5]; a neonatal bilirubin management app that improves management [6]; and a commercially successful app, sold through multiple app stores, that dynamically generates patient-specific medication instructions [7]. Because apps access data using the SMART on FHIR API, they can be installed or deleted at any institution, independent of the underlying EHR, akin to smartphone apps. The “without special effort” clause should necessitate that the public API enable this capability without purchasing paywalled features from an EHR vendor. An interoperable apps–based economy could drive down costs, support improved public health surveillance and response [8], bring machine learning to the point of care [9], and accelerate genome-informed medicine [10,11].

Smartphone apps are distributed through app stores. The Apple App store and Google Play store, for example, are large “one-stop shopping” markets, enable turnkey installation, and offer customer review aggregation, technical and security reviews, and payment processing. As such, they are a unique and invaluable channel to deliver technology to an end user. However, innovators are charged as much as 30% of revenue, and Apple has found itself under intense scrutiny for controlling both the platform (the iPhone) and the marketplace (the App Store) [12]. Since EHR vendors have taken a similar tack [13], the final ONC rule wisely prohibits anticompetitive behaviors, including offering different service terms to similar apps, noncompete and exclusivity clauses, and intellectual property–transfer requirements. The major EHR vendors now have app store equivalents, like the Epic App Orchard, Cerner App Gallery, or AllScripts Application Store, as do new entrant companies constructing proprietary health care app ecosystems separate from the EHR vendors.

## Risks of Misaligned Incentives and Anticompetitive Behavior

Despite information blocking provisions, business practices could stifle innovation and reduce choice. First of all, there is an issue of pricing and fees. Under the HITECH Act, the United States has already invested US $48 billion toward the promotion of EHR adoption. Because most EHR products are based on pre-internet software, the opportunity to layer on a modern infrastructure is essential for progress. The final ONC rule does not quantify permitted API fees, but allows for recouping fees “reasonably incurred” by the EHR vendor. How costs will be passed down is unclear. Health system leaders would be right to ask how much they should be expected to pay to get their own data out of EHR products they have already purchased. Patients too have already effectively paid for their data, through insurance premiums, taxes, or directly out of pocket. The Apple and Google app stores are proven to inspire developers to produce millions of apps. It is far from clear to us that the EHR marketplaces would lead to similar much-needed innovation in health care.

Secondly, we are concerned that EHR vendors may limit functionality and data availability across the public APIs and instead shift app connections to higher functioning proprietary APIs. The rule would permit an EHR vendor to profit from value-added services, as long as those services are not necessary for developing and deploying software that uses the API. It is extremely important that the public, regulated APIs give patients, providers, and innovators robust functionality. One issue is that the rule only requires a subset of data elements to be exposed through public APIs — the US Core Data for Interoperability (USCDI). Another is that the rule focuses on reading data from the EHR, rather than writing data back to the EHR. If the government does not expand the USCDI rapidly enough, or stalls on advancing a write capability, the proprietary APIs may outpace open, public, standardized APIs. An app written once would require a different version and set of agreements for each marketplace, analogous to a need to create a different version of a web page for every different web browser. Furthermore, EHR vendors may circumvent the spirit of the Cures Act and ONC rule by levying prohibitive revenue-sharing schemes for apps that access a modified version of the public API, charge for favorable placement in EHR-associated app stores, or limit important usability features, for example, requiring nonpreferred apps to frequently “log back in” to receive updated data. Health care organizations may be unaware of these innovation-blocking behaviors and powerless to stop them.

Thirdly, the ONC final rule applies to certified API developers — for now, these are predominantly EHR vendors. However, the rule may not apply to emerging secondary platforms that use the public API to move data into a proprietary system with a proprietary API, for example solutions built on iOS or Android. We could end up exchanging one closed ecosystem (EHRs) for another (secondary platforms), which will further segment the market, block innovation, and limit physician and patient choice.

Finally, assessing real-world implementation of the Cures Act will be challenging. Infractions of the “without special effort” provision could emerge through interpretations of the final rule, be hidden behind business contracts and nondisclosure agreements, or be promulgated through hesitancy to address EHR vendor business practices. Initial API usage has not been high [14,15], and much of it is attributable to Apple Health Records on iPhone alone. The good news is that the slow pace allows time to shape the unfolding ecosystem as the rule’s provisions take effect over the next 2 years. Both manual and automated processes are needed to measure key provisions of the final rule [16] and assess whether the Cures Act has produced a robust apps economy where an app written once can run widely throughout health care.

## Conclusion

While the Cures Act and ONC final rule place guardrails around information blocking, a truly interoperable plug-and-play app ecosystem is far from predetermined. There is ample room for “innovation blocking” even by vendors who are regulatorily compliant. Measurement of progress toward an open app ecosystem and additional regulation are needed to ensure return on the massive investment in national digital infrastructure.

## Abbreviations

API: application programming interface
EHR: electronic health record
FHIR: Fast Healthcare Interoperability Resource
ONC: Office of the National Coordinator for Health Information Technology
SMART: Substitutable Medical Applications, Reusable Technologies
USCDI: US Core Data for Interoperability
